# Supplementary material for: Patterns of Intron Gain and Loss in Fungi
Source: PLoS Biol. 2004 Nov 30;2(12):e422. doi: 10.1371/journal.pbio.0020422 (PMC532390; doi:10.1371/journal.pbio.0020422)
Supplement: Table S1 — Also available at http://genes.mit.edu/NielsenEtAl/. (4.3 MB ZIP). [file pbio.0020422.st001.zip › NielsenEtAl/html/424.html]

AN6265.1.NCU01502.1.MG01100.1.FG04404.1


```
 CLUSTAL W (1.82) Multiple Sequence Alignments - Introns Inserted


Sequence 1: NCU01502.1	554 aa
Sequence 2: FG04404.1	552 aa
Sequence 3: MG01100.1	556 aa
Sequence 4: AN6265.1	535 aa
Alignment Length: 557 aa
Number Identitical Residues: 267 aa
Alignment Score (without introns) 14278


MG01100.1 	METLERKSTGSIPGLKEQANGALAVRDARTSLTSKADEAKKRRQKQAQRQYGRGAGVDVR
NCU01502.1	METTDRAEISLSR--AEQPSGAVAINNVRSEFRSKAEANKIRKVKAAEKSYGRGRKIDVK
FG04404.1 	MEVET-VNTTISK--VRD-NGATAVEGARMELVPRSDRERARRIREAHKAYGRGKKVNLK
AN6265.1  	----------------------MADSAPTKAIVPTKQRKENKRLIEAQKKYGRGKAVHTH
          	                       *       : .  :  . ::   *.: ****  :. :

MG01100.1 	SIKNKKLRRTLGQLEQKYKDAALEAKDAEILHENEAGFLEPEGEL~ERTYKTRQDEITEG
NCU01502.1	NVKDKKLKRNLTNLEEKYQTATLKAKEAEILLENAGGFLEAEHEL0ERTYKVRQEDIVSE
FG04404.1 	AIKDKKLRSNMKRLESKYEDAALKAKDAEILLENTSGFLEAEDEL~ERTYKVRQDDISSG
AN6265.1  	SVRDKKLRSNLQAVEEKYKNAALRAKDAEILLEHEAGFLEPEGEM~ERTYRVRQDEIAAS
          	 :::***: .:  :*.**: *:*.**:**** *: .****.* *: ****:.**::*   

MG01100.1 	VAVETAQKRFDLSLDGLGPYLCDYTRNGRELLIGGRKGHVATFDWREGKLGCEIQLGETV
NCU01502.1	VNIEVAQKKFDLKLDQLGPYICDFSRNGRDLILAGRKGHVATMDWRDGKLGCELQLGETI
FG04404.1 	VAVGTAQKRFDLNLDQLGPYQFDYSRNGRDLLLGGRKGHVATMDWREGKLGCELQLNETI
AN6265.1  	VGIETAKKKFELKLEDFGPYRADYTRNGRELLLAGRKGHIATMDWRNGKLGCELHLRETV
          	* : .*:*:*:*.*: :***  *::****:*::.*****:**:***:******::* **:

MG01100.1 	RDVKWLHNNQYFAVAQKKTVYLYDRNGVELHNLRKHINVTHMEFLPYHFLLCTA0SDTGM
NCU01502.1	RDVKWLHNNQYFAVAQKKYTYIYDSQGVELHCLRKHVEVSHLEFLPYHFLLATL~GTNGQ
FG04404.1 	RDVKWLHNNQYFAVAQKKYVYIYDHNGVELHTLRKHQEVSHMEFLPYHYLLATI0GSVGF
AN6265.1  	RDARWLHNNQYFAVAQKKSVYIYDSQGVELHNLDRIVEPCFLEFLPYHFLLASA0QMSGH
          	**.:************** .*:** :***** * :  :  .:******:**.:     * 

MG01100.1 	LKYQDTSTGQIVSEVASKLGPTQSLVQNPWNAILHMGHNNGTVTLWSPNSSDPLVKLLAH
NCU01502.1	LKYQDTSTGQIVTEIATKLGTPVSMTQNPWNAILHVGHQNGTVTLWSPNSSEPLVKLLAH
FG04404.1 	LKYQDTSTGQLVAEIPTRLGQPCALKQNPWNAILHVGHQNGTVTLWSPNSSDPLVKLLAH
AN6265.1  	LKYTDTSTGQLVAEIPTKVGAPTSLAQNPWNAIMHVGHQNGTVTLWSPNTQTPLVKALVH
          	*** ******:*:*:.:::* . :: *******:*:**:**********:. **** *.*

MG01100.1 	KGPVRSLAIDREGRYMVSTGQDSRMAVWDIRMFKEVNNYFTRTPASSVAISDSGLTAVGW
NCU01502.1	RGPVRSMAVDREGRYMVTTGQDCKMAVWDIRMFKEVNNYFTRAPASSVAISDTGLTAVGW
FG04404.1 	RGPVRDVAVDREGRYMVSTGQDQKMAVWDLRMLREVNSYFTRQPASSVAISDTGLTAIGW
AN6265.1  	RGPVRSMAIDRQGRYMVSTGQDLKMQVWDIRMYREVHSYSCHQPGAAVSISDRGLTAVGW
          	:****.:*:**:*****:**** :* ***:** :**:.*  : *.::*:*** ****:**

MG01100.1 	GTHTTIWKGLFDKNAATQEKVQSPYLTWGGEGRRVERVRWCPFEDVLGIGHDGGFSSILV
NCU01502.1	GTRTTVWKGLFSKEKPVQEKVQSPYMTWGGEGKRVERVRWCPFDDVLGVGHSEGFSSLII
FG04404.1 	GTQTTIWKGLFDKNAPVQEKVQSPYMAWGGEGKRIERVRWCPFEDVLGIGHDSGFSSIIV
AN6265.1  	GTQVSVWRGLFDAATADVGKVQSPYMSWGGDGQRIENLRWAPYEDILGVAHDKGFASIIV
          	**:.::*:***.   .   ******::***:*:*:*.:**.*::*:**:.*. **:*:::

MG01100.1 	PGAGEANFDALEINPYETVKQRQESEVKSLLNKLQPEMIALDPNFVGNLDLRSEAQRRAE
NCU01502.1	PGAGEANFDALEVNPYETKKQRQEGEVKQLLNKLAPEMIALDPNFIGTLDLRSAKQREAD
FG04404.1 	PGAGEANYDALEVNPFETAKQRQESEVKGLLNKLQPDMIALDPNYIGTLDLRSEKQRRAE
AN6265.1  	PGAGEPNFDALEVNPYENPKQRQEAEVRALLNKLQPGMISLDPNFVGKVDTVSDKRSREE
          	*****.*:****:**:*. *****.**: ***** * **:****::*.:*  *  : . :

MG01100.1 	RDLDAPPEDIADEIRNRMRGKNSALKKYLRKQRKKNVIDEKRLKIEEMWKEQQDKRDERH
NCU01502.1	KDLDAGPPDLEEEMRNRARGKNSALKKYLRKQKKKNIIDEKRLKAEEMYKQMQEKNGERH
FG04404.1 	KDLDAPAADIAEEIRKRARGKNGALKKYLRKQRKKNIIDEKRMHVDEIWNEQQVKKNKKE
AN6265.1  	KDLDRRPEDTIEKLKNRGRGRNSALRKYLRKKGRRNVIDEKLLKAQALHKERSARAKERY
          	:***  . *  :::::* **:*.**:*****: ::*:**** :: : : :: . :  :: 

MG01100.1 	KAQQEELGPALARFARKE-
NCU01502.1	KEKVAELGPTLARFARKE-
FG04404.1 	LEAEADLGPALSRFAKKE-
AN6265.1  	RAEREELGPALARFAKKDI
          	     :***:*:***:*:
```
